# The quality of life in Ecuadorian patients with multiple sclerosis and neuromyelitis optica spectrum disorder and their association with anxiety, depression, fatigue, and disability

**DOI:** 10.3389/fneur.2026.1721735

**Published:** 2026-05-01

**Authors:** Edgar Patricio Correa-Díaz, Ruth Jimbo-Sotomayor, Patricio Merino-Aguilera

**Affiliations:** 1 Pontificia Universidad Católica del Ecuador, Quito, Ecuador; 2 Neurology Department, Carlos Andrade Marín Specialties Hospital, Quito, Ecuador; 3 Hospital General de México Dr. Eduardo Liceaga, Mexico City, Mexico

**Keywords:** Beck, EDSS, Hamilton, MSQOL-54, multiple sclerosis, neuromyelitis optica spectrum disorder, quality of life

## Abstract

**Background:**

MS and NMOSD are chronic demyelinating diseases related to demyelinating attacks on the central nervous system which limit quality of life and predisposes patients to developing anxiety and depression which, in turn, leads to a worsening of their disease.

**Methods:**

It is a cross-sectional and analytical study which was carried out at Hospital Carlos Andrade Marín (HCAM) in Quito, Ecuador. We included 151 patients in this study. The MSQOL-54 Scale Spanish version was used. The Hamilton Anxiety Scale, the Beck Depression Inventory, and the Modified Fatigue Impact Scale were applied. Pearson’s correlation was performed to establish an association between two continuous variables. This study was approved by the ethics committee of the HCAM.

**Results:**

In our study, we found that the quality of life was more affected in NMOSD than MS patients (*p* < 0.005). NMOSD patients presented higher EDSS scores in relation to MS (*p* < 0.05). Anxiety and depression were frequent in both diseases. Mild anxiety was frequent in MS, whereas moderate and severe anxiety was common in NMOSD patients. Mild depression predominated in both pathologies. Fatigue was more frequent in MS than NMOSD (*p* < 0.01). We found that the younger NMOSD patients had lower scores of QoL. We found correlations between, degree of disability, fatigue, anxiety, and depression with the QoL of our patients in both MS and NMOSD.

**Conclusion:**

The presence of anxiety, depression, fatigue and a higher degree of disability was associated with a poorer quality of life in both diseases.

## 1 Introduction

Multiple sclerosis (MS) and neuromyelitis optica spectrum disorder (NMOSD) are autoimmune chronic inflammatory diseases of the central nervous system (CNS) that cause demyelination and axonal damage (1). For many years, NMOSD was considered a severe variant of MS. However, they have different pathological and radiological characteristics (2). In Ecuador, both pathologies are not common; MS has a prevalence of 4 cases per 100,000 inhabitants (3), while NMOSD prevalence and incidence are still unknown, and only one observational study has been published so far (4).

MS and NMOSD are associated with cognitive impairment, motor and sensory disorders, fatigue, pain, and sexual problems which limit the quality of life (QoL) and predispose the patients to anxiety, depression, and worsening of their disease (5). The World Health Organization (WHO) defines Health-Related Quality of Life (HRQoL) as “the individual’s perception of their position in life within the cultural context and value system in which they live, considering their goals, expectations, standards, and concerns” (6). The assessment of QoL is important in the context of health since it allows us to more precisely measure the emotional and economic impact that chronic diseases have on the population (7).

The QoL has been widely studied in MS patients. However, few studies have evaluated the QoL in NMOSD patients; the majority of studies come from North America, Europe, and Asia (8–14) and only one study has reported QoL in NMOSD patients from Latin America (2). Therefore, the aim of this study is to describe the QoL in NMOSD and MS patients from Ecuador and to compare the QoL between the two groups of patients. Additionally, it seeks to measure the impact of anxiety, depression, fatigue, and disability on the QoL in both diseases.

## 2 Methods

A cross-sectional and descriptive study was carried out at Carlos Andrade Marín Hospital in the city of Quito, Ecuador, which is a referral center for patients with MS and NMOSD. Currently, 60% of patients with MS and 70% of patients with NMOSD diagnosed in Ecuador are attended at this center. We enrolled MS and NMOSD patients who met the 2017 McDonald criteria and 2015 international consensus criteria, respectively (15, 16). The study was performed between March and June 2025.

### 2.1 Instruments

A questionnaire was developed to collect socio-demographic data of patients. Additionally, clinical data were obtained from the hospital medical records. Neurological disability was assessed using the EDSS (expanded disability status scale) score (17). AQP4 was tested by cell-based assay (CBA) in all NMOSD patients (18).

The MSQOL-54 Scale (Multiple Sclerosis Quality of Life-54) Spanish version was used to study QoL which has good internal consistency since it presents a Cronbach’s alpha ranging from 0.75 to 0.96 (19). The MSQOL-54 Scale was created based on the 36-Item Short Form Health Survey (SF-36). In addition, 18 items were added. MSQOL-54 has been used in Mexican patients with MS in order to measure QoL (20) while the SF-36 has been used previously to measure QoL in NMOSD patients from LATAM (2) and has been validated in the LATAM population (21, 22). So, we believe that it is possible to use the MSQOL-54 to measure QoL in Ecuadorian patients with both diseases.

The MSQOL-54 Scale consists of 54 questions which evaluate 14 subscales: 1. Physical Health 2. Performance limitations due to physical problems 3. Performance limitations due to emotional problems 4. Pain 5. Emotional well-being 6. Energy 7. Health perceptions 8. Social function 9. Cognitive function 10. Health problems 11. Sexual function 12. Changes in health 13. Satisfaction with sexual function and 14. General quality of life from which two main final scores are obtained: the physical and mental health composite. The physical and mental health composite questionnaires yield scores between 0 and 100, considering that 100 represents the highest QoL level (best) and 0 is the lowest QoL level (worst). It is considered that with a score of 60 or less, the patients have the worst QoL (23).

In order to analyze the impact of anxiety on the QoL, we used the Hamilton Anxiety Scale (24) which evaluates 14 points: anxious mood, tension, fears, insomnia, intellect, depressed mood, muscular discomfort, sensory discomfort, cardiovascular, respiratory, gastrointestinal, genitourinary, autonomic symptoms, and interview behavior with a total score ranging from 0 to 56. A score of 0 to 5 indicates absence of anxiety, 6 to 14 mild anxiety, and equal to or higher than 15 moderate/severe anxiety.

The Beck Depression Inventory (BDI-II) was also used to assess the degree of depression in patients with demyelinating diseases and its impact on the QoL. This scale consists of 21 questions with a total score ranging from 0 to 40. A score of 0 to 9 points means that the patient does not present depression, 10 to 18 points is mild depression, 19 to 29 points moderate depression, and equal to or higher than 30 points is severe depression (51). The Hamilton Anxiety Scale and BDI-II scales have been previously validated in Ecuador (25, 26). Finally, The Modified Fatigue Impact Scale (MFIS) was applied in order to determine fatigue in both diseases. MFIS has been validated in LATAM populations (27).

This study was approved by the ethics committee of the Carlos Andrade Marín Hospital. All participants signed an informed consent form before data collection.

### 2.2 Statistical analysis

All data analysis was carried out through the statistical program Microsoft Excel and the software SPSS for Windows version 25.0. Data were analyzed in a descriptive manner by measuring central tendency (mean) and dispersion (confidence interval (CI) and standard deviation (SD)). We used proportions for the analysis of categorical variables. Comparisons between categorical variables were made by chi-square contingencies tables and the Fisher exact test of significance. Student’s *t*-test and ANOVA were used for the univariate comparison when data showed statistical normality whereas Mann–Whitney U or Kruskal–Wallis H test were used when the distribution was non-normal. Finally, Pearson’s correlation was performed to establish an association between two continuous variables. The significance level was established as *p* < 0.05. Due to the exploratory nature of the study, no sample size calculation was made.

## 3 Results

A total of 190 patients with MS and NMOSD were identified, of whom 151 agreed to participate and were included in the study (117 with MS and 34 with NMOSD). The female sex was predominant in both diseases, being more frequent in NMOSD than in MS (85.29% vs. 70.94% respectively). It is interesting to note that 11% of MS patients were Caucasian vs. 0% of the NMOSD patients. At the onset of the disease, MS patients had the first symptom almost a decade earlier than NMOSD patients (32.3 vs. 39.8 years respectively; *p* < 0.05). NMOSD patients had worse disability than MS patients according to EDSS (4.23 ± 1.73 vs. 2.5 ± 1.95 *p* < 0.05). Anti-AQP-4 antibodies were positive in 67.65% of NMOSD patients. The demographic and clinical characteristics of the sample are presented in Table 1.

**Table 1:** Socio-demographic and clinical characteristics of patients with MS and NMOSD.

Variable	MS (*n* = 117)	NMOSD (*n* = 34)
	*n* (%)	*n* (%)
Sex		
Male	34 (29.06%)	5 (14.71%)
Female	83 (70.94%)	29 (85.29%)
Ethnic group		
Mestizo Indigenous	103 (88.03%)	31 (91.18%)
African-American	0	0
Caucasian	0	1 (2.94%)
Montubio	14 (11.97%)	0
Other	0	1 (2.94%)
	0	1 (2.94%)
Age		
Mean (± SD)	42.3 (12.71)	49 (13.24)
Median (IQR)	42 (16–76)	48.5 (22–79)
Current age (years)		
<18	3 (2.56%)	0
18 to 30	21 (17.95%)	2 (5.88%)
31 to 40	29 (24.79%)	5 (14.71%)
41 to 50	31 (26.50%)	13 (38.24%)
51 to 60	23 (19.66%)	9 (26.47%)
> 61	10 (8.55%)	5 (14.71%)
Marital status		
Single	46 (39.32%)	5 (14.71%)
Married	47 (40.17%)	26 (76.47%)
Widowed	3 (2.56%)	1 (2.94%)
Divorced	16 (13.68%)	1 (2.94%)
Common-law marriage	5 (4.27%)	1 (2.94%)
Educational level		
Primary	2 (1.71%)	6 (17.65%)
High-school	26 (22.22%)	10 (29.41%)
University	89 (76.07%)	18 (52.94%)
None	0	0
Symptom onset (years)		
< 2	14 (11.97%)	3 (8.82%)
2 to 5	18 (15.38%)	10 (29.41%)
5 to 10	27 (23.08%)	10 (29.41%)
> 10 years	58 (49.57%)	11 (32.35%)
EDSS		
Mean (± SD)	2.5 (1.95)	4.2 (1.73)
Median (IQR)	2 (0–7.5)	4 (0–7.5)
Level of disability (EDSS)		
None or mild disability (0–3.5)	76 (64.96%)	6 (17.65%)
Moderate disability (4–6)	34 (29.06%)	21 (61.76%)
Severe disability (>6.5)	7 (5.98%)	7 (20.59%)
Phenotype of MS		
RRMS	101 (86.32%)	–
SPMS	6 (5.13%)	–
PPMS	5 (4.27%)	–
CIS	4 (3.42%)	–
RIS	1 (0.85%)	–
AQP4 antibody in NMOSD		
Positive	–	23 (67.65%)
Negative	–	9 (26.47%)
Unknown	–	2 (5.88%)

MS, multiple sclerosis; NMOSD, neuromyelitis optica spectrum disorder; SD, standard deviation; IQR, interquartile range, EDSS, expanded disability status scale; RRMS, relapsing remitting MS; SPMS, secondary progressive MS; PPMS, primary progressive MS; CIS, clinically isolated syndrome; RIS, radiologically isolated syndrome; AQP4, aquaporin-4 antibodies.

Regarding QoL in NMOSD, we found lower scores of QoL in comparison with MS (*p* < 0.05). We found a significant difference between MS and NMOSD patients in both physical (62.05 ± 20.2 and 45.91 ± 17.8, respectively, *p* < 0.004) and mental health composites (59.71 ± 22.18 vs. 51.23 ± 19.02, respectively, *p* = 0.045). In addition, no statistical differences were found in the subscale scores in the physical and mental health composite in both diseases according to gender (Table 2).

**Table 2:** MSQOL-54 results in patients with MS and NMOSD.

MS				
MSQOL-54	Total	Women	Men	p*
	(*n* = 117)	(*n* = 83)	(*n* = 34)	
	Mean (± SD)	Mean (± SD)	Mean (± SD)	
Overall physical health	62.05 (20.28)	61.3 (20.7)	63.8 (19.2)	0.546
Physical health	67.69 (29.5)	66.2 (29.9)	71.3 (28.3)	0.397
Health perception	58.29 (20.09)	57,17 (20.4)	61.03 (19.3)	0.348
Energy	54.9 (19.7)	53.9 (20.16)	57.2 (18.7)	0.411
Physical limitations	51.7 (43.07)	51.2 (44.5)	52.9 (39.7)	0.844
Pain	68.09 (25.4)	66.8 (26.6)	71.07 (22.6)	0.419
Sexual function	70.08 (30.7)	68.9 (31.5)	72.7 (28.9)	0.544
Social function	66.8 (26.18)	67.16 (25.4)	66.17 (28.19)	0.853
Health concerns	61.03 (27.3)	61.7 (27.5)	59.2 (27.2)	0.658
Overall mental health	59.7 (22.1)	59.9 (22.8)	59.1 (20.7)	0.875
Quality of life	59.9 (18.7)	59.3 (19.5)	61.4 (16.6)	0.594
Emotional well-being	63.4 (19.8)	63.8 (20.4)	62.4 (18.4)	0.733
Emotional limitations	52.4 (43.6)	52.2 (44.2)	52.9 (42.7)	0.935
Cognitive function	62.5 (26.9)	63.5 (26.5)	60.15 (28.16)	0.537

NMOSD				
MSQOL-54	Total	Women	Men	p*
	(*n* = 34)	(*n* = 29)	(*n* = 5)	
	Mean (± SD)	Mean (± SD)	Mean (± SD)	
Overall physical health	45.9 (17.8)	45.3 (18.3)	49.4 (16.2)	0.638
Physical health	48.09 (32.07)	47.9 (33.5)	49.0 (24.8)	0.946
Health perception	46.9 (14.2)	46.7 (14.8)	48 (10.9)	0.857
Energy	46.3 (16.9)	46.2 (17.15)	47.2 (17.2)	0.906
Physical limitations	29.4 (32.8)	28.4 (34.5)	35 (22.3)	0.687
Pain	47.2 (22.8)	45.9 (23.9)	54.3 (14.9)	0.458
Sexual function	56.3 (37.1)	59.4 (35.8)	38.3 (43.9)	0.246
Social function	45.8 (22.7)	43.9 (22.3)	56.6 (24.5)	0.256
Health concerns	49.7 (27.6)	46.9 (27.6)	66 (23.5)	0.157
Overall mental health	51.2 (19.01)	49.02 (18.1)	64.01 (20.7)	0.104
Quality of life	52.15 (19.5)	51.6 (19.2)	55.3 (23.2)	0.701
Emotional well-being	56.2 (18.9)	55.3 (17.4)	61.6 (28.3)	0.502
Emotional limitations	41.17 (40.2)	36.7 (39.18)	66.6 (40.8)	0.127
Cognitive function	57.9 (26.3)	55.3 (27.15)	73 (15.2)	0.17

^*^Student-t for independent samples.

MS, multiple sclerosis; NMOSD, multiple sclerosis spectrum disorder; MSQOL-54, Quality of life-54; SD, standard deviation; *p*, *p*-value.

Anxiety was less frequent in MS in comparison with NMOSD patients (64% vs. 82%, respectively, p = 0.045). Fifty-nine percent of NMOSD patients had moderate/severe anxiety in contrast with 31% of MS patients (*p* = 0.004). Depression was also more common in NMOSD in comparison with MS patients (82.35% vs. 55.6%, respectively, *p* < 0.05). Mild and moderate depression were more frequent in NMOSD than in MS (*p* = 0.001). There was no difference in severe depression in both NMOSD and MS patients (*p* = 0.06) (Table 3). In MS patients, a negative and significant correlation was found between physical and mental health and depression scores (*r* = −0.59 and −0.68, respectively, *p*-value <0.01). Similarly, a negative and significant correlation was found between physical and mental health and anxiety scores (*r* = −0.61 and −0.73, respectively, *p*-value <0.01). In NMOSD patients, there was a negative and significant correlation between QoL and anxiety scores in both physical and mental health composite (*r* = −0.38 and −0.36, respectively, *p*-value <0.05). However, in NMOSD patients with depression, there was a negative correlation in physical score (*r* = −0.38 *p*-value <0.05) but not with mental score (*r* = 0.21 *p*-value >0.05) (Table 4).

**Table 3:** Analysis of the quality of life and anxiety and depression in patients with MS and NMOSD.

	MSQOL-54			
Anxiety	MS		NMOSD	
	Physical health	Mental health	Physical health	Mental health
	Mean (SD±)	Mean (SD±)	Mean (SD±)	Mean (SD±)
No anxiety	74.29 (18.19)	76.54 (16.72)	62.20 (13.44)	71.99 (15.68)
Mild	62.55 (18.38)	61.54 (18.27)	55.88 (11.14)	62.56 (12.28)
Moderate/Severe	47.24 (14.26)	38.07 (10.73)	37.04 (15.88)	40.47 (14.00)
p* <0.001 < 0.003			0.001 < 0.002	

Depression	Physical health	Mental health	Physical health	Mental health
	Mean (SD±)	Mean (SD±)	Mean (SD±)	Mean (SD±)
No depression	75.08 (16.16)	73.27 (17.82)	60.43 (11.10)	65.99 (16.41)
Mild	55.76 (14.18)	55.17 (17.70)	45.59 (16.76)	52.65 (13.01)
Moderate	48.90 (20.30)	44.46 (18.41)	40.91 (12.78)	37.28 (12.23)
Severe	37.99 (12.57)	28.65 (11.91)	20.80 (6.55)	29.75 (24.69)
p* <0.001 < 0.006			0.001 < 0.001	

*One-way ANOVA.

MS, multiple sclerosis; NMOSD, multiple sclerosis spectrum disorder; MSQOL-54, Quality of life-54; SD, standard deviation.

**Table 4:** Correlation of disability, anxiety, and depression according to type of disease and quality of life in patients with MS and NMOSD.

Variable	Multiple sclerosis (*n* = 117)			Neuromyelitis optica (*n* = 34)		
	Disability	Anxiety	Depression	Disability	Anxiety	Depression
	*r* Mean (SD**+/−**)	*r* Mean (SD**±**)	*r* Mean (SD**±**)	*r* Mean (SD**±**)	*r* Mean (SD**±**)	*r* Mean (SD**±**)
MSQoL physical health (Global)	−0.556** 53.6 (18.7)	−0.613** 61.3 (16.9)	−0.592** 54.4 (15.8)	−0.471** 49.1 (14.2)	−0.389* 51.7 (13.4)	−0.384* 41.9 (11.8)
Physical health	−0.659** 49.2 (23.4)	−0.253** 67.4 (29.3)	−0.282** 62.3 (28.7)	−0.206 52.2 (24.3)	−0.573** 55.8 (27.7)	−0.475** 43.6 (26.8)
Health perception	−0.350** 52.1 (19)	−0.441** 57.7 (18)	−0.430** 53.3 (15.9)	−0.061 48.4 (12.7)	−0.551** 52.17 (11.4)	−0.657** 44.2 (12.1)
Energy	−0.255** 51.3 (19)	−0.521** 54.3 (17.1)	−0.572** 49.06 (16.8)	−0.365* 47.5 (13.7)	−0.303 50.6 (16.05)	−0.405* 42.9 (13.8)
Physical limitations	−0.471** 38.8 (39.1)	0.541** 50.3 (36.7)	−0.534** 36.9 (30.1)	−0.162 36.7 (25.6)	−0.436** 35.5 (32.8)	−0.432* 24.8 (23.6)
Pain	−0.420** 60 (26.6)	−0.383** 67.5 (24)	−0.330** 63.2 (25.3)	−0.228 49.4 (20.3)	−0.593** 51.6 (21.3)	−0.456** 44.7 (19.7)
Sexual function	−0.137 69.8 (32.9)	−0.434** 69.3 (28.4)	−0.406** 61.8 (33.1)	0.029 57.4 (36.3)	−0.508** 62.5 (34.6)	−0.483** 49.03 (28.8)
Social function	−0.454** 58.3 (26.7)	−0.602** 66.02(22.1)	−0.530** 57.1 (21.4)	−0.327 50.9 (20.5)	−0.591** 49.3 (20.5)	−0.665** 43.1 (23.1)
Health concerns	−0.265** 57.8 (26.8)	−0.528** 60.1 (23.4)	−0.473** 51.5 (23.1)	−0.371* 52.3 (26.4)	−0.664** 58.1 (22.9)	−0.670** 44.01 (16.5)
MSQoL mental health (Global)	−0.348** 55.2 (22.2)	−0.732** 58.7 (15.2)	−0.658** 50.4 (16.4)	−0.100 53.9 (17.8)	−0.368* 58.3 (13.9)	−0.257 46.4 (16.5)
Emotional limitations	−0.333** 44.5 (44.4)	−0.629** 50.7 (33.6)	−0.534** 38.9 (35.6)	0.077 46.03 (41.8)	−0.644** 51.8 (36.3)	−0.689** 39.6 (40.6)
Emotional well-being	−0.157 62 (19)	−0.645** 62.6 (15.5)	−0.651** 55.4 (16.5)	−0.007 57.8 (15.1)	−0.516** 61.2 (17.4)	−0.534** 50.9 (14.7)
Cognitive function	−0.201* 59.6 (31)	−0.581** 61.6 (22.7)	−0.466** 54.4 (23.7)	−0.191 58.8 (26.7)	−0.627** 63.5 (23.2)	−0.696** 50.6 (23.6)
Quality of life	−0.446** 52.9 (15.6)	−0.466** 59.4 (16.5)	−0.466** 53.4 (17.1)	−0.132 55.05 (16.1)	−0.717** 58.1 (16.08)	−0.705** 46.6 (16.1)

Pearson’s correlation, *p < 0.05, **p < 0.01. MS, multiple sclerosis; NMOSD, multiple sclerosis spectrum disorder; MSQOL-54, Quality of life-54; SD, standard deviation; EDSS, expanded disability status scale.

According to disability measure by EDSS, MS patients had a negative correlation in both physical and mental health composites (*r* = −0.55 and −0.34 respectively, *p* < 0.05). In NMOSD, there was a negative correlation between disability and QoL but only in physical component (*r* = −0.47, p < 0.05) (Table 4).

Fatigue was more frequent in MS than NMOSD (43% vs. 21% *p* < 0.01). There was an association between QoL scores and fatigue scores in both the physical (*p* = 0.001) and mental (0.048) scores in MS patients, but we did not find this finding in NMOSD patients (*p* = 0.2).

## 4 Discussion

In our study, MS patients had better quality of life in relation to NMOSD patients. The average of the physical and mental scores in patients with MS were higher in comparison with NMOSD patients (*p* < 0.005). The most affected components were the emotional, physical, social, and sexual. It was observed that the quality of life was affected to the same extent in both genders, and there was a negative correlation between, disability, anxiety, depression, and fatigue scores with Qol. These findings are very similar to those found by Carnero Contentti et al. (2) who demonstrated that NMOSD patients scored significantly lower in general health when they were compared with MS patients.

Regarding ethnicity, in our study the majority of MS patients were mestizo and 11% were Caucasian, we did not identify Amerindians with MS. In LATAM the largest ethnic group is mestizo which is a result of complex racial admixture of Caucasian Europeans, Amerindians, and Black Sub-Sahara Africans. In this region the prevalence of MS is heterogeneous and depends on the presence of White or Amerindian population, being higher in areas such as Buenos Aires y Montevideo (25–38 cases per 100.000 inhabitants) in which there are more White population and lower in areas such as Ecuador and Bolivia (1–5 cases per 100.000 inhabitants) in which the population is mainly Mestizo and Amerindian (11, 28). One possible explanation of this difference in prevalence comes from a genetic study with Mexicans patients with MS, this study has showed that European ancestry was a risk factor for develop MS while indigenous ancestry seems to confer protection (29). In contrast, in our study, NMOSD patients were mestizos and we did not identify White people with NMOSD. A recent study has demonstrated that NMOSD susceptibility is highest in Black people and lowest in White people (30), this results can explain why in our study no white people with NMOSD were founded but more epidemiological and genetic studies from LATAM in both diseases are necessary in order to determine whether this increased susceptibility is due to shared genetic ancestry.

Several studies have demonstrated the association of MS and the risk of divorce. In our study 13% of our MS patients were divorced this finding is lower in comparison with a study of 371 MS Spanish patients which showed that 31% of them were divorced/separated after an average disease duration of 10 years (31). Another study has demonstrated that the incidence of divorced in male patients with MS was 21% but there was not significant difference in female MS patients (32). Interesting marital status has an influence on QoL as was demonstrated in a study in which married patients had higher scores in physical and mental composite (33). In NMOSD, one recent study from China has demonstrated that the percentage of divorce in NMOSD patients was very low (1.6%) this result is very similar to our cohort (2.9%). Male patients had less probability of get married but in our study we did not make this analysis (34). In comparison with MS, marital status in NMOSD has received little attention and more studies are necessary.

A predominance of mild disability in MS was found with an average EDSS of 2.5, which was surprising since a systematic review found that the mean EDSS in LATAM populations is higher, between 4.5 to 6 (35). On the other hand, we found that the EDSS in our NMOSD patients was 4.2, and it is very similar if we compare this finding with EDSS average in LATAM of 4.3 (36). A recent study has showed that in NMOSD patients the initial symptoms and neurological signs at admission were more severe than MS. (37) Therefore, NMOSD is a more disabling disease in comparison with MS. We found that disability was associated with a significant impairment of the QoL in both MS and NMOSD patients. This result is very similar to other reports. Thus, in LATAM, Carnero Contentti et al. (2) showed in 53 patients with NMOSD and 100 with MS that a higher EDSS, longer disease duration, and the presence of relapses in the previous year were significantly associated with a lower overall health score in NMOSD (*p* < 0.05). Beekman et al. (10) have demonstrated in 193 patients with NMOSD that quality of life was mainly affected when the patient had a greater degree of disability. Barzegar et al. (9) also found that NMOSD patients who presented a higher EDSS, longer disease duration, fatigue, anxiety, and depression were associated with a worse quality of life (*p* < 0.01).

In our study, the prevalence of anxiety was high in both diseases; mild anxiety was more frequent in MS patients while moderate/severe anxiety was more frequent in NMOSD patients. These results are very similar to those presented in other reports. Thus, one study in MS patients demonstrated a high prevalence of anxiety in this disease (70%) (38). Additionally, anxiety is also common in NMOSD; a meta-analysis found that the prevalence of anxiety was 40% (39). In our study, we found a negative correlation between anxiety and QoL in both diseases. Previous studies have demonstrated similar results. For example, a study conducted in 223 patients with MS showed a negative correlation between anxiety and QoL (40). In NMOSD, an Argentine study found that mild and moderate anxiety was associated with lower QoL scores (2).

Regarding depression, we found a high prevalence of this disease in both MS and NMOSD. Moreover, NMOSD patients presented lower depression scores in relation to MS patients (*p* = 0.001). Depression is common in MS patients; the lifetime prevalence is between 40 and 60% (52). In LATAM, an Argentinean study found that depression was present in 45% of NMOSD patients with a predominance of mild depression (41). Depression can affect the QoL of patients with demyelinating diseases. We found that the presence of depression was associated with lower QoL scores in patients with MS in both physica and mental components. A prospective study showed that depression was a predictor of low QoL in MS patients since depression can exacerbate clinical problems such as pain, fatigue, and anxiety (52). In NMOSD patients, we found a negative correlation between depression with QoL. This result is very similar to those reported in other studies. A German study which included 166 patients with NMOSD demonstrated that depression was associated with a low level of QoL (8, 42, 43).

Additionally, we showed that NMOSD patients with chronic pain had lower QoL scores on the pain subscale and in the physical component in relation to MS. A Chinese study demonstrated that NMOSD patients with chronic pain had a greater inability to work and participate in social activities (12, 44). It is worth mentioning that in our study, in both diseases, the patients presented a low prevalence of fatigue when we compare our data with results from a systematic review in which the prevalence of fatigue in MS patients was between 75 to 90% (45). A study in NMOSD patients also found a high prevalence of fatigue in this disease, around 71.4% (46). The presence of fatigue in our patients with MS was associated with low QoL scores in both the physical (*p* = 0.001) and mental (0.048) components, but we did not find a relationship between fatigue and quality of life in NMOSD patients (*p* = 0.2). This finding is not similar to those reported by Barzegar et al. (9) in which there was a significant association between male NMOSD patients and fatigue in cognitive category (*p* < 0.001). One possible explanation of this difference is that in our study we did not performed this analysis between fatigue and Qol in NMOSD patients by sex and cognitive category. In addition, the pathophysiology of fatigue in NMOSD is not clear and the evidence of their association with NMOSD is limited. Therefore, more studies on the association of fatigue and NMOSD are necessary specially in LATAM populations (47–50).

Our study has limitations that can be highlighted. In this project, there were sample losses since not all the patients participated in the study; the number of patients is small compared to studies carried out in other countries. There are incomplete medical records that prevented obtaining certain data. As it is a cross-sectional study, it is difficult to evaluate the sample over time; the surveys can overestimate the results in some patients; however, digital media containing specific answers were used to reduce these drawbacks. It has not been possible to establish a single score in international literature that accurately shows a breakpoint between good and poor quality of life, except for lower scores on the scale which are associated with lower QoL. There are few studies in Ecuador that help to compare QoL in these diseases; there is no study on quality of life carried out specifically for patients with NMOSD in Ecuador that helps us compare the results of this study with previous findings and establish conclusions.

Finally, in our study, we found correlations between age, degree of disability, fatigue, anxiety, and depression with the QoL of our patients in both MS and NMOSD. It is very clear that the QoL was more affected in NMOSD than MS patients. There was no difference in the QoL by sex. It is very interesting to highlight that in both diseases, the female sex and mestizo predominated. NMOSD patients are younger than MS patients. NMOSD patients presented higher EDSS scores in relation to MS. Anxiety and depression were frequent in both diseases; however, mild anxiety was frequent in MS, and it contrasts with anxiety in NMOSD patients which was moderate to severe. Mild depression predominated in both pathologies. These results are valuable since they have been considered predictors of poor prognosis in these pathologies in previous studies. There are no similar studies in Ecuador and there are few studies in LATAM populations. For this reason, the present study allows us to understand the impact of MS and NMOSD on the quality of life in LATAM populations in which MS and NMOSD are diseases with a low prevalence.

## Data Availability

The original contributions presented in the study are included in the article/supplementary material, further inquiries can be directed to the corresponding author.
